# Draft genome sequence of *Lactiplantibacillus plantarum* BS25 isolated from a local fermented rice-shrimp mixture from the Philippines

**DOI:** 10.1128/mra.00808-23

**Published:** 2024-01-05

**Authors:** Zachary B. Lara, Mia Beatriz C. Amoranto, Francisco B. Elegado, Leslie Michelle M. Dalmacio, Marilen P. Balolong

**Affiliations:** 1 Department of Biochemistry and Molecular Biology, College of Medicine, University of the Philippines, Manila, Philippines; 2 National Institute of Molecular Biology and Biotechnology, University of the Philippines, Los Baños, Philippines; 3 Department of Biology, College of Arts and Sciences, University of the Philippines, Manila, Philippines; Indiana University, Bloomington, Indiana, USA

**Keywords:** probiotics, lactic acid bacteria, Philippines, fermented food, illumina, genomes

## Abstract

The draft genome sequence of *Lactiplantibacillus plantarum* BS25, previously isolated from a fermented rice-shrimp mixture (*balao-balao*) in the Philippines, was analyzed. The genome contains 3,264,139 bp (44.62% GC%), and a total of 3,069 predicted coding sequences, 2 rRNAs, and 52 tRNAs.

## ANNOUNCEMENT

*Lactiplantibacillus plantarum* (formerly *Lactobacillus plantarum*) is a Gram-positive lactic acid-producing bacteria that has a high ecological and metabolic adaptability in a wide range of environmental niches, including fermented foods (1). In the Philippines, *L. plantarum* has been isolated from several local fermented foods (2). *L. plantarum* BS25, previously isolated from *balao-balao* (fermented rice-shrimp mixture) (3), harbors bacteriocinogenic and anticholesterolemic properties (3, 4) and is cytotoxic against colorectal cancer cells (5). Furthermore, it has inhibitory properties against methicillin-sensitive and -resistant *Staphylococcus aureus* (6). Owing to its potential application as probiotics, its safety and biofunctional properties should be assessed using whole-genome sequencing. Here, we report the draft genome sequence of *L. plantarum* BS25.

BS25 was previously isolated from *balao-balao*, a traditional Filipino condiment, obtained from a wet market in Tarlac, Philippines (3). BS25 was grown in de Man, Rogosa, and Sharpe (HiMedia Laboratories, Germany) at 30°C for 24 h. Genomic DNA (gDNA) was extracted using the Wizard gDNA Purification Kit (Promega Corporation, WI, USA) according to manufacturer’s protocol, with minor modifications. Library preparation was performed using the TruSeq DNA Nano Kit (Illumina, USA), and sequencing was conducted using an Illumina MiSeq instrument (2 × 150 cycles) at the Philippine Genome Center (Quezon City, Philippines). A total of 3,516,166 reads, with an average length of 150 bp, were generated. Quality control was performed using fastp 0.23.2 (7). Contigs were assembled using Spades 3.15.5 (8), with the—sc and—careful presets specified—a combination producing the least amount of mis-assemblies. Scaffold quality was assessed using Quast 5.2.0 (9) and CheckM 1.1.6 (10), while predicted genes were annotated using the NCBI Prokaryotic Genome Annotation Pipeline (PGAP) 6.5 (11).

The draft genome of BS25 contains 3,264,139 bp (N50 = 229,189 bp) in 44 contigs, a GC content of 44.62%, and completeness of 99.38%. The strain shared an average nucleotide identity of 99.96% with *L. plantarum* DOMLa (CP004406.1), the closest related strain identified to date. A total of 3,069 predicted coding sequences, 2 rRNAs, and 52 tRNAs were identified in the chromosome.

## Data Availability

The draft genome assembly and raw reads are deposited in NCBI GenBank under the accession numbers GCA_031032805.1 and SRS18543367, respectively.
